# Heptasodium tetra­aluminium tetra­kis­(diphosphate) orthophosphate, Na_7_Al_4_(P_2_O_7_)_4_(PO_4_)

**DOI:** 10.1107/S1600536811041729

**Published:** 2011-10-22

**Authors:** Dan Zhao

**Affiliations:** a Department of Physics and Chemistry, Henan Polytechnic University, Jiaozuo, Henan 454000, People’s Republic of China

## Abstract

The asymmetric unit of title compound contains three Na^+^, one Al^3+^, three P^5+^ and eight O^2−^ atoms, with one Na^+^ atom lying on a twofold rotation axis and one Na^+^ and one P^5+^ atom on fourfold rotoinversion axes. The fundamental building units of the title structure are isolated PO_4_ tetra­hedra, AlO_6_ octa­hedra and P_2_O_7_ groups, which are further inter­locked by corner-sharing O atoms, forming a three-dimensional framework structure. The Na^+^ atoms are located within the cavities of the framework, showing coordination numbers of 4, 6 and 7.

## Related literature

For isotypic structures, see: Rochère *et al.* (1985 ▶); Stus *et al.* (2001 ▶).

## Experimental

### 

#### Crystal data

Na_7_Al_4_(P_2_O_7_)_4_(PO_4_)
*M* *_r_* = 1059.58
Tetragonal, 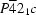

*a* = 14.054 (3) Å
*c* = 6.1718 (16) Å
*V* = 1219.1 (4) Å^3^
*Z* = 2
Mo *K*α radiation
μ = 1.06 mm^−1^
*T* = 296 K
0.15 × 0.05 × 0.05 mm

#### Data collection

Rigaku Saturn70 CCD diffractometer
Absorption correction: multi-scan (*ABSCOR*; Higashi, 1995 ▶) *T*_min_ = 0.857, *T*_max_ = 0.949
5562 measured reflections
1347 independent reflections
1287 reflections with *I* > 2σ(*I*)
*R*_int_ = 0.024

#### Refinement

*R*[*F* ^2^ > 2σ(*F* ^2^)] = 0.017
*wR*(*F* ^2^) = 0.047
*S* = 1.08
1347 reflections
118 parameters
Δρ_max_ = 0.22 e Å^−3^
Δρ_min_ = −0.29 e Å^−3^
Absolute structure: Flack (1983 ▶), 540 Friedel pairs
Flack parameter: −0.04 (9)

### 

Data collection: *CrystalClear* (Rigaku, 2004 ▶); cell refinement: *CrystalClear*; data reduction: *CrystalClear*; program(s) used to solve structure: *SHELXS97* (Sheldrick, 2008 ▶); program(s) used to refine structure: *SHELXL97* (Sheldrick, 2008 ▶); molecular graphics: *DIAMOND* (Brandenburg, 2004 ▶); software used to prepare material for publication: *SHELXTL* (Sheldrick, 2008 ▶).

## Supplementary Material

Crystal structure: contains datablock(s) I, global. DOI: 10.1107/S1600536811041729/wm2530sup1.cif

Structure factors: contains datablock(s) I. DOI: 10.1107/S1600536811041729/wm2530Isup2.hkl

Additional supplementary materials:  crystallographic information; 3D view; checkCIF report

## Figures and Tables

**Fig. 1.**
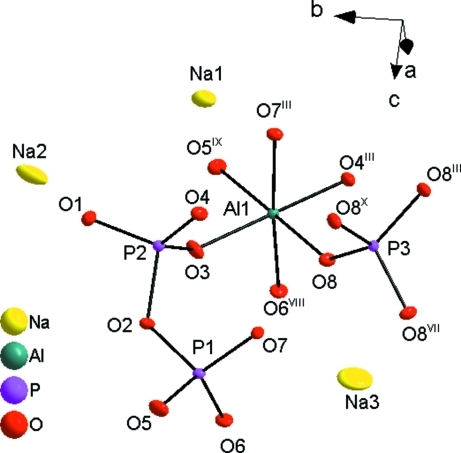
The expanded asymmetric unit of Na7(AlP2O7)4PO4 showing the coordination environments of the P and Al atoms. The displacement ellipsoids are drawn at the 50% probability level. [Symmetry codes: (iii) -y + 1, x, -z + 1; (vii) -x + 1, -y + 1, z; (viii) -y + 1, x, -z + 2; (ix) x - 1/2, -y + 3/2, -z + 3/2; (x) y, -x + 1, -z + 1.]

**Fig. 2.**
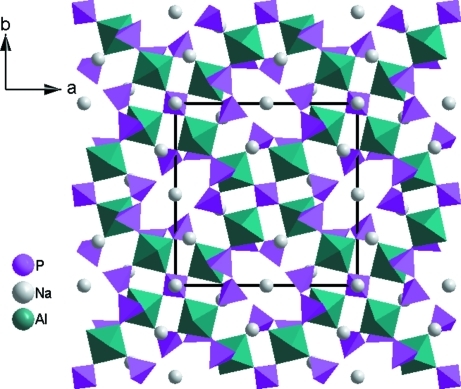
View of the crystal structure of Na7(AlP2O7)4PO4. PO4 and AlO6 units are given in the polyhedral representation.

## supplementary crystallographic information

### Comment

Metal phosphates possessing open-framework structures with defined tunnels have been extensively investigated for their structural diversity, properties, and potential applications in shape-selective catalysis, adsorbents, ion exchangers, and molecular sieves. Among them, a series of isotypic ortho-diphosphates Na_7_(MP_2_O_7_)_4_PO_4_ (M = Al, Cr, Fe) (Rochère et al., 1985) and Na_7_(InP_2_O_7_)_4_PO_4_ (Stus et al., 2001) were synthesized, and their ion exchange and conductivity properties studied. However, for compound Na_7_(AlP_2_O_7_)_4_PO_4_, a detailed crystal structure analysis has not been reported so far. In this work, the synthesis and results of the crystal structure refinement of this compound is reported. In comparison with the unit cell parameters reported by Rochère from X-ray powder data (a = 14.046 (3), c = 6.169 (2) Å; Rochère et al., 1985), the determined unit cell parameters from the single crystal X-ray study are slighty larger.

As shown in Figs 1 and 2, the structure of the title compound consists of a three-dimensional framework of isolated PO_4_ tetrahedra, AlO_6_ octahedra and P_2_O_7_ groups, the conformation of the latter more eclipsed than staggered. The sodium cations are located in sites within cavities in the framework, exhibiting coordination numbers of 7 (Na1), 6 (Na2) and 4 (Na3). There are three crystallography distinct P atoms in the structure of the title compound. P1 and P2 atoms are located in general positions and their corresponding P1O_4_ and P2O_4_ tetrahedra are connected by the bridging O5 atom to form a P_2_O_7_ group, which is further linked to four AlO_6_ octahedra. P3 atoms are located on 4 axes, forming isolated P3O_4_ tetrahedra which are further connected to four AlO_6_ octahedra. The P3O_4_ tetrahedra are regular with a P—O bond length of 1.5351 (18) Å, while the P–O distances in the P_2_O_7_ group are irregular, showing the characteristic variance of smaller P–O_terminal_ bonds (1.4862 (14) to 1.5199 (14) Å) and larger P–O_bridging_ bonds (1.6097 (14) and 1.6284 (15) Å) as typically observed for diphosphate unit. The title structure differs in their P–O–P bridging angle of the diphosphate group and the average metal–O distances from those of the isotypic congeners. For the Al compound, the interatomic distances in the MO_6_ octahedron are decreasing (Fe–O_6_ 1.968–2.021 Å, InO_6_ 2.091–2.146 Å, AlO_6_ 1.8391 (15)–1.9278 (16) Å), as expected from the smaller ionic radius of Al^3+^ comparerd to Fe^3+^ and In^3+^. With a decrease of the unit-cell parameters a trend in a likewise decreasing P–O–P bridging angle of the diphosphate groups is observed: (Na_7_(FeP_2_O_7_)_4_PO_4_: 136.6 (3)°; Na_7_(InP_2_O_7_)_4_PO_4_: 136.7 (3)°, Na_7_(AlP_2_O_7_)_4_PO_4_: 127.92 (9)°.

### Experimental

The finely ground reagents Na_2_CO_3_, Al_2_O_3_ and NH_4_H_2_PO_4_ were mixed in the molar ratio Na: Al: P = 2: 1: 8, were placed in a Pt crucible, and heated at 673 K for 4 h. The mixture was then re-ground and heated at 1173 K for 20 h, then cooled to 673 K at a rate of 3 K h^-1^, and finally quenched to room temperature. A few colorless crystals of the title compound with prismatic shape were obtained.

### Refinement

The highest peak in the difference electron density map equals to 0.22 e/Å^3^ at the distance of 0.63 Å from O4 site while the deepest hole equals to -0.29 e/Å^3^ at the distance of 0.68 Å from the P3 site.

### Crystal data

**Table:**

Na_7_Al_4_(P_2_O_7_)_4_(PO_4_)	*D*_x_ = 2.887 Mg m^−^^3^
*M**_r_* = 1059.58	Mo *K*α radiation, λ = 0.71073 Å
Tetragonal, *P*42_1_*c*	Cell parameters from 3720 reflections
Hall symbol: P -4 2n	θ = 2.9–27.5°
*a* = 14.054 (3) Å	µ = 1.06 mm^−^^1^
*c* = 6.1718 (16) Å	*T* = 296 K
*V* = 1219.1 (4) Å^3^	Prism, colourless
*Z* = 2	0.15 × 0.05 × 0.05 mm
*F*(000) = 1040	

### Data collection

**Table:**

Rigaku Saturn70 CCD diffractometer	1347 independent reflections
Radiation source: fine-focus sealed tube	1287 reflections with *I* > 2σ(*I*)
Graphite Monochromator	*R*_int_ = 0.024
Detector resolution: 14.6306 pixels mm^-1^	θ_max_ = 27.5°, θ_min_ = 3.2°
ω scans	*h* = −18→17
Absorption correction: multi-scan (*ABSCOR*; Higashi, 1995)	*k* = −18→16
*T*_min_ = 0.857, *T*_max_ = 0.949	*l* = −7→7
5562 measured reflections	

### Refinement

**Table:**

Refinement on *F*^2^	Primary atom site location: structure-invariant direct methods
Least-squares matrix: full	Secondary atom site location: difference Fourier map
*R*[*F*^2^ > 2σ(*F*^2^)] = 0.017	*w* = 1/[σ^2^(*F*_o_^2^) + (0.0297*P*)^2^ + 0.278*P*] where *P* = (*F*_o_^2^ + 2*F*_c_^2^)/3
*wR*(*F*^2^) = 0.047	(Δ/σ)_max_ = 0.001
*S* = 1.08	Δρ_max_ = 0.22 e Å^−^^3^
1347 reflections	Δρ_min_ = −0.29 e Å^−^^3^
118 parameters	Absolute structure: Flack (1983), 540 Friedel pairs
0 restraints	Flack parameter: −0.04 (9)

### Special details

**Table:**

Geometry. All e.s.d.'s (except the e.s.d. in the dihedral angle between two l.s. planes) are estimated using the full covariance matrix. The cell e.s.d.'s are taken into account individually in the estimation of e.s.d.'s in distances, angles and torsion angles; correlations between e.s.d.'s in cell parameters are only used when they are defined by crystal symmetry. An approximate (isotropic) treatment of cell e.s.d.'s is used for estimating e.s.d.'s involving l.s. planes.
Refinement. Refinement of *F*^2^ against ALL reflections. The weighted *R*-factor *wR* and goodness of fit *S* are based on *F*^2^, conventional *R*-factors *R* are based on *F*, with *F* set to zero for negative *F*^2^. The threshold expression of *F*^2^ > σ(*F*^2^) is used only for calculating *R*-factors(gt) *etc*. and is not relevant to the choice of reflections for refinement. *R*-factors based on *F*^2^ are statistically about twice as large as those based on *F*, and *R*- factors based on ALL data will be even larger.

### Fractional atomic coordinates and isotropic or equivalent isotropic displacement parameters (Å^2^)

**Table:**

	*x*	*y*	*z*	*U*_iso_*/*U*_eq_	
Na1	0.42251 (6)	0.75865 (7)	0.09314 (15)	0.0200 (2)	
Na2	0.5000	1.0000	0.3156 (2)	0.0268 (3)	
Na3	0.5000	0.5000	1.0000	0.0411 (6)	
Al1	0.32097 (4)	0.62225 (4)	0.63747 (9)	0.00558 (13)	
P1	0.62752 (3)	0.74207 (3)	0.84990 (8)	0.00573 (11)	
P2	0.46166 (3)	0.79988 (3)	0.60041 (8)	0.00656 (11)	
P3	0.5000	0.5000	0.5000	0.00535 (19)	
O1	0.42717 (10)	0.89936 (10)	0.5600 (2)	0.0117 (3)	
O2	0.54197 (10)	0.81323 (10)	0.7882 (2)	0.0109 (3)	
O3	0.38751 (10)	0.73621 (10)	0.7038 (2)	0.0128 (3)	
O4	0.51085 (9)	0.75581 (10)	0.4067 (2)	0.0104 (3)	
O5	0.71136 (9)	0.80522 (10)	0.8791 (2)	0.0129 (3)	
O6	0.59683 (10)	0.69263 (10)	1.0575 (2)	0.0113 (3)	
O7	0.63718 (10)	0.66852 (9)	0.6721 (2)	0.0087 (3)	
O8	0.43148 (9)	0.55247 (10)	0.6522 (2)	0.0100 (3)	

### Atomic displacement parameters (Å^2^)

**Table:**

	*U*^11^	*U*^22^	*U*^33^	*U*^12^	*U*^13^	*U*^23^
Na1	0.0224 (4)	0.0242 (5)	0.0133 (4)	−0.0060 (3)	−0.0034 (4)	0.0031 (4)
Na2	0.0280 (7)	0.0405 (9)	0.0118 (6)	−0.0216 (6)	0.000	0.000
Na3	0.0561 (9)	0.0561 (9)	0.0111 (9)	0.000	0.000	0.000
Al1	0.0049 (2)	0.0063 (3)	0.0055 (3)	0.0010 (2)	0.0002 (2)	0.0000 (2)
P1	0.0056 (2)	0.0069 (2)	0.0047 (2)	−0.00024 (16)	0.00004 (17)	0.00036 (18)
P2	0.0064 (2)	0.0068 (2)	0.0064 (2)	0.00048 (16)	−0.00085 (19)	−0.00024 (18)
P3	0.0053 (3)	0.0053 (3)	0.0054 (4)	0.000	0.000	0.000
O1	0.0133 (7)	0.0101 (6)	0.0117 (8)	0.0030 (6)	−0.0018 (6)	0.0007 (6)
O2	0.0106 (7)	0.0123 (7)	0.0097 (7)	0.0043 (5)	−0.0043 (6)	−0.0034 (6)
O3	0.0139 (7)	0.0127 (7)	0.0118 (7)	−0.0066 (6)	0.0023 (6)	−0.0033 (6)
O4	0.0086 (6)	0.0138 (7)	0.0088 (6)	0.0027 (5)	0.0000 (5)	−0.0019 (6)
O5	0.0101 (7)	0.0166 (7)	0.0121 (8)	−0.0056 (5)	−0.0004 (6)	−0.0012 (6)
O6	0.0149 (7)	0.0134 (7)	0.0055 (7)	−0.0024 (5)	0.0008 (6)	0.0021 (5)
O7	0.0119 (7)	0.0076 (7)	0.0065 (7)	0.0015 (5)	−0.0005 (5)	0.0007 (5)
O8	0.0081 (6)	0.0135 (7)	0.0084 (6)	0.0039 (5)	0.0000 (6)	−0.0014 (5)

### Geometric parameters (Å, °)

**Table:**

Na1—O4	2.2998 (17)	P1—O7	1.5136 (14)
Na1—O1^i^	2.3993 (17)	P1—O6	1.5199 (14)
Na1—O3^ii^	2.4730 (18)	P1—O2	1.6097 (14)
Na1—O7^iii^	2.5789 (17)	P2—O1	1.5006 (14)
Na1—O6^ii^	2.6291 (18)	P2—O4	1.5134 (14)
Na1—O2^ii^	2.6363 (18)	P2—O3	1.5145 (15)
Na1—O6^iii^	2.9419 (18)	P2—O2	1.6284 (15)
Na2—O1^iv^	2.3072 (16)	P3—O8^iii^	1.5341 (14)
Na2—O1	2.3072 (17)	P3—O8^x^	1.5341 (14)
Na2—O1^i^	2.3532 (17)	P3—O8	1.5341 (14)
Na2—O1^v^	2.3532 (17)	P3—O8^vii^	1.5341 (14)
Na2—O2^i^	2.6957 (15)	O1—Na2^xi^	2.3532 (17)
Na2—O2^v^	2.6957 (15)	O1—Na1^xii^	2.3993 (17)
Na3—O8^vi^	2.4655 (16)	O2—Na1^xiii^	2.6363 (18)
Na3—O8	2.4655 (16)	O2—Na2^xi^	2.6957 (15)
Na3—O8^vii^	2.4655 (16)	O3—Na1^xiii^	2.4730 (18)
Na3—O8^viii^	2.4655 (16)	O4—Al1^x^	1.9210 (15)
Al1—O8	1.8391 (15)	O5—Al1^xiv^	1.8500 (15)
Al1—O5^ix^	1.8500 (15)	O6—Al1^vi^	1.9258 (16)
Al1—O3	1.8993 (16)	O6—Na1^xiii^	2.6291 (18)
Al1—O4^iii^	1.9210 (15)	O6—Na1^x^	2.9419 (18)
Al1—O6^viii^	1.9258 (16)	O7—Al1^x^	1.9278 (16)
Al1—O7^iii^	1.9278 (16)	O7—Na1^x^	2.5789 (17)
P1—O5	1.4862 (14)		
			
O4—Na1—O1^i^	99.33 (6)	O3—Al1—O6^viii^	89.69 (7)
O4—Na1—O3^ii^	157.36 (7)	O4^iii^—Al1—O6^viii^	86.10 (7)
O1^i^—Na1—O3^ii^	90.92 (6)	O8—Al1—O7^iii^	92.43 (7)
O4—Na1—O7^iii^	77.49 (5)	O5^ix^—Al1—O7^iii^	87.07 (6)
O1^i^—Na1—O7^iii^	129.35 (6)	O3—Al1—O7^iii^	94.82 (7)
O3^ii^—Na1—O7^iii^	111.27 (6)	O4^iii^—Al1—O7^iii^	89.49 (6)
O4—Na1—O6^ii^	63.97 (5)	O6^viii^—Al1—O7^iii^	175.36 (7)
O1^i^—Na1—O6^ii^	117.88 (6)	O5—P1—O7	115.13 (8)
O3^ii^—Na1—O6^ii^	93.38 (6)	O5—P1—O6	113.31 (8)
O7^iii^—Na1—O6^ii^	106.00 (5)	O7—P1—O6	108.92 (9)
O4—Na1—O2^ii^	105.20 (6)	O5—P1—O2	104.47 (8)
O1^i^—Na1—O2^ii^	74.83 (5)	O7—P1—O2	108.65 (8)
O3^ii^—Na1—O2^ii^	58.00 (5)	O6—P1—O2	105.76 (8)
O7^iii^—Na1—O2^ii^	155.46 (6)	O1—P2—O4	113.43 (9)
O6^ii^—Na1—O2^ii^	56.60 (5)	O1—P2—O3	113.46 (8)
O4—Na1—O6^iii^	123.33 (6)	O4—P2—O3	113.91 (8)
O1^i^—Na1—O6^iii^	131.42 (6)	O1—P2—O2	103.58 (8)
O3^ii^—Na1—O6^iii^	59.00 (5)	O4—P2—O2	107.01 (8)
O7^iii^—Na1—O6^iii^	52.62 (5)	O3—P2—O2	104.19 (8)
O6^ii^—Na1—O6^iii^	102.31 (6)	O8^iii^—P3—O8^x^	104.48 (11)
O2^ii^—Na1—O6^iii^	110.46 (5)	O8^iii^—P3—O8	112.02 (6)
O1^iv^—Na2—O1	98.36 (9)	O8^x^—P3—O8	112.02 (6)
O1^iv^—Na2—O1^i^	166.32 (7)	O8^iii^—P3—O8^vii^	112.02 (6)
O1—Na2—O1^i^	84.54 (5)	O8^x^—P3—O8^vii^	112.02 (6)
O1^iv^—Na2—O1^v^	84.54 (5)	O8—P3—O8^vii^	104.48 (11)
O1—Na2—O1^v^	166.32 (7)	P1—O2—P2	127.92 (9)
O1^i^—Na2—O1^v^	95.80 (8)	P1—O2—Na1^xiii^	97.23 (7)
O1^iv^—Na2—O2^i^	109.82 (5)	P2—O2—Na1^xiii^	91.90 (7)
O1—Na2—O2^i^	75.12 (5)	P1—O2—Na2^xi^	138.90 (8)
O1^i^—Na2—O2^i^	57.84 (5)	P2—O2—Na2^xi^	90.30 (6)
O1^v^—Na2—O2^i^	116.61 (6)	Na1^xiii^—O2—Na2^xi^	95.69 (5)
O1^iv^—Na2—O2^v^	75.12 (5)	P2—O3—Al1	138.27 (10)
O1—Na2—O2^v^	109.82 (5)	P2—O3—Na1^xiii^	101.37 (7)
O1^i^—Na2—O2^v^	116.61 (6)	Al1—O3—Na1^xiii^	114.51 (7)
O1^v^—Na2—O2^v^	57.84 (5)	P2—O4—Al1^x^	135.59 (9)
O2^i^—Na2—O2^v^	172.79 (8)	P2—O4—Na1	114.27 (8)
O8^vi^—Na3—O8	139.29 (4)	Al1^x^—O4—Na1	109.28 (7)
O8^vi^—Na3—O8^vii^	139.29 (4)	P1—O5—Al1^xiv^	169.27 (10)
O8—Na3—O8^vii^	58.94 (7)	P1—O6—Al1^vi^	144.77 (10)
O8^vi^—Na3—O8^viii^	58.94 (7)	P1—O6—Na1^xiii^	100.00 (7)
O8—Na3—O8^viii^	139.29 (4)	Al1^vi^—O6—Na1^xiii^	97.24 (6)
O8^vii^—Na3—O8^viii^	139.29 (4)	P1—O6—Na1^x^	76.46 (6)
O8—Al1—O5^ix^	178.73 (7)	Al1^vi^—O6—Na1^x^	96.37 (6)
O8—Al1—O3	91.33 (7)	Na1^xiii^—O6—Na1^x^	160.22 (7)
O5^ix^—Al1—O3	87.55 (7)	P1—O7—Al1^x^	131.02 (9)
O8—Al1—O4^iii^	92.68 (6)	P1—O7—Na1^x^	89.48 (7)
O5^ix^—Al1—O4^iii^	88.48 (6)	Al1^x^—O7—Na1^x^	131.83 (7)
O3—Al1—O4^iii^	173.98 (7)	P3—O8—Al1	139.13 (10)
O8—Al1—O6^viii^	86.36 (7)	P3—O8—Na3	98.29 (7)
O5^ix^—Al1—O6^viii^	94.23 (6)	Al1—O8—Na3	122.17 (7)

Symmetry codes: (i) *y*−1/2, *x*+1/2, *z*−1/2; (ii) *x*, *y*, *z*−1; (iii) −*y*+1, *x*, −*z*+1; (iv) −*x*+1, −*y*+2, *z*; (v) −*y*+3/2, −*x*+3/2, *z*−1/2; (vi) *y*, −*x*+1, −*z*+2; (vii) −*x*+1, −*y*+1, *z*; (viii) −*y*+1, *x*, −*z*+2; (ix) *x*−1/2, −*y*+3/2, −*z*+3/2; (x) *y*, −*x*+1, −*z*+1; (xi) −*y*+3/2, −*x*+3/2, *z*+1/2; (xii) *y*−1/2, *x*+1/2, *z*+1/2; (xiii) *x*, *y*, *z*+1; (xiv) *x*+1/2, −*y*+3/2, −*z*+3/2.
